# Disrupted in schizophrenia 1 (DISC1) inhibits glioblastoma development by regulating mitochondria dynamics

**DOI:** 10.18632/oncotarget.13290

**Published:** 2016-11-11

**Authors:** Xingchun Gao, Yajing Mi, Na Guo, Zhifang Hu, Fengrui Hu, Dou liu, Lei Gao, Xingchun Gou, Weilin Jin

**Affiliations:** ^1^ Institute of Basic Medical Sciences and Shaanxi key Laboratory of ischemic Cardiovascular Disease, Xi’an Medical University, Xi’an 710021, P. R. China; ^2^ Institute of Nano Biomedicine and Engineering, Department of Instrument Science and Engineering, Key Lab. for Thin Film and Microfabrication Technology of Ministry of Education, School of Electronic Information and Electronic Engineering, Shanghai Jiao Tong University, Shanghai 200240, P. R. China; ^3^ National Centers for Translational Medicine, Shanghai Jiao Tong University, Shanghai 200240, P. R. China

**Keywords:** glioblastoma, DISC1, mitochondria dynamics, self-renewal, Drp1

## Abstract

Glioblastoma(GBM) is one of the most common and aggressive malignant primary tumors of the central nervous system and mitochondria have been proposed to participate in GBM tumorigenesis. Previous studies have identified a potential role of Disrupted in Schizophrenia 1 (DISC1), a multi-compartmentalized protein, in mitochondria. But whether DISC1 could regulate GBM tumorigenesis via mitochondria is still unknown. We determined the expression level of DISC1 by both bioinformatics analysis and tissue analysis, and found that DISC1 was highly expressed in GBM. Knocking down of DISC1 by shRNA in GBM cells significantly inhibited cell proliferation both *in vitro* and *in vivo*. In addition, down-regulation of DISC1 decreased cell migration and invasion of GBM and self renewal capacity of glioblastoma stem-like cells. Furthermore, multiple independent rings or spheres could be observed in mitochondria in GBM depleted of DISC1, while normal filamentous morphology was observed in control cells, demonstrating that DISC1 affected the mitochondrial dynamic. Dynamin-related protein 1 (Drp1) was reported to contribute to mitochondrial dynamic regulation and influence glioma cells proliferation and invasion by RHOA/ ROCK1 pathway. Our data showed a significant decrease of Drp1 both in mRNA and protein level in GBM lack of DISC1, indicating that DISC1 maybe affect the mitochondrial dynamic by regulating Drp1. Taken together, our findings reveal that DISC1 affects glioblastoma cell development via mitochondria dynamics partly by down regulation of Drp1.

## INTRODUCTION

Glioblastoma is one of the most common primary tumors of the central nervous system. Comprehensive treatment including operation combined with radiotherapy and/or chemotherapy is always used in order to delay the recurrence and prolong survival. However, the estimated median survival of glioma patients is still very short, which is approximately 1–2 years [1]. Previous studies have identified many factors influencing the effectiveness of glioma therapies, in which the rapid and aggressive tumor growth might be the most important. Therefore, suppressing glioblastoma cell proliferation and inhibiting cell migration would be the foremost therapeutic strategy.

Mitochondria are dynamic organelles whose size and subcellular distribution are constantly changing, and this feature is essential for maintaining mitochondria related biological activityies, such as cell growth, division, death, aging and diseases [2–4]. Mitochondrial dynamics are under the regulation of two opposing processes: fusion and fission. Fusion is regulated by the Mitofusion1/2 and Optic Atrophy 1(OPA1). Fission is regulated by dynamin-related protein 1 (Drp1). Unbalanced mitochondrial fusion and fission has been proposed to contribute to tumorigenesis by influencing mitochondrial functions and following synthesis of DNA and protein, apoptosis, autophagy, ROS production [3, 5–7]. In HCC cells, increased mitochondrial fission was observed and proved to be essential in cell growth^6^. The imbalance of mitochondrial fusion and fission in glioma U251MG cells treated with anticancer drug of 4EGI-1, resulted in mitochondrial dysfunction with mitochondrial fragmentation [8]. In addition, Xie *et al*. showed that mitochondrial morphology was a regulatory switch for differentiation of glioma stem cells [9]. All the data showed a critical role of mitochondrial dynamics in tumorigenesis.

The Disrupted in Schizophrenia 1 (DISC1) gene, was originally identified to be linked to schizophrenia, and the (1; 11) (q42; q14.3) translocation allele of the DISC1 gene cosegregated with symptoms related to schizophrenia, bipolar in a large Scottish pedigree [10]. More recently, functional studies revealed that DISC1 exerted other roles in the process of neurodevelopment, including neurite outgrowth, neuronal migration, neurogenesis and cAMP signaling [11, 12]. But there is no report about that whether DISC1 plays a role in tumorigenesis. It had been reported that DISC1 was localized in mitochondria of cultured cortical neurons [13], and over expression of DISC1 produced ring-like structures of mitochondria in some cells, suggesting that DISC1 might be involved in the regulation of mitochondrial dynamics and further contribute to tumorigenesis [14].

In the present study, we found notable increase of DISC1 protein level in human glioblastoma cells and inhibition of DISC1 by shRNAs reduced glioblastoma cell proliferation, migration, invasion and stem cell self-renewal. Furthermore, we characterized the molecular basis of DISC1 in glioblastoma carcinogenesis. In all, our data suggested that DISC1 could affect mitochondria dynamics of glioblastoma cell via promoting Drp1 expression. Thus, our research may provide a novel direaction for the anticancer therapies.

## RESULTS

### Up-regulation of DISC1 expression in human glioblastoma

**Figure 1:**
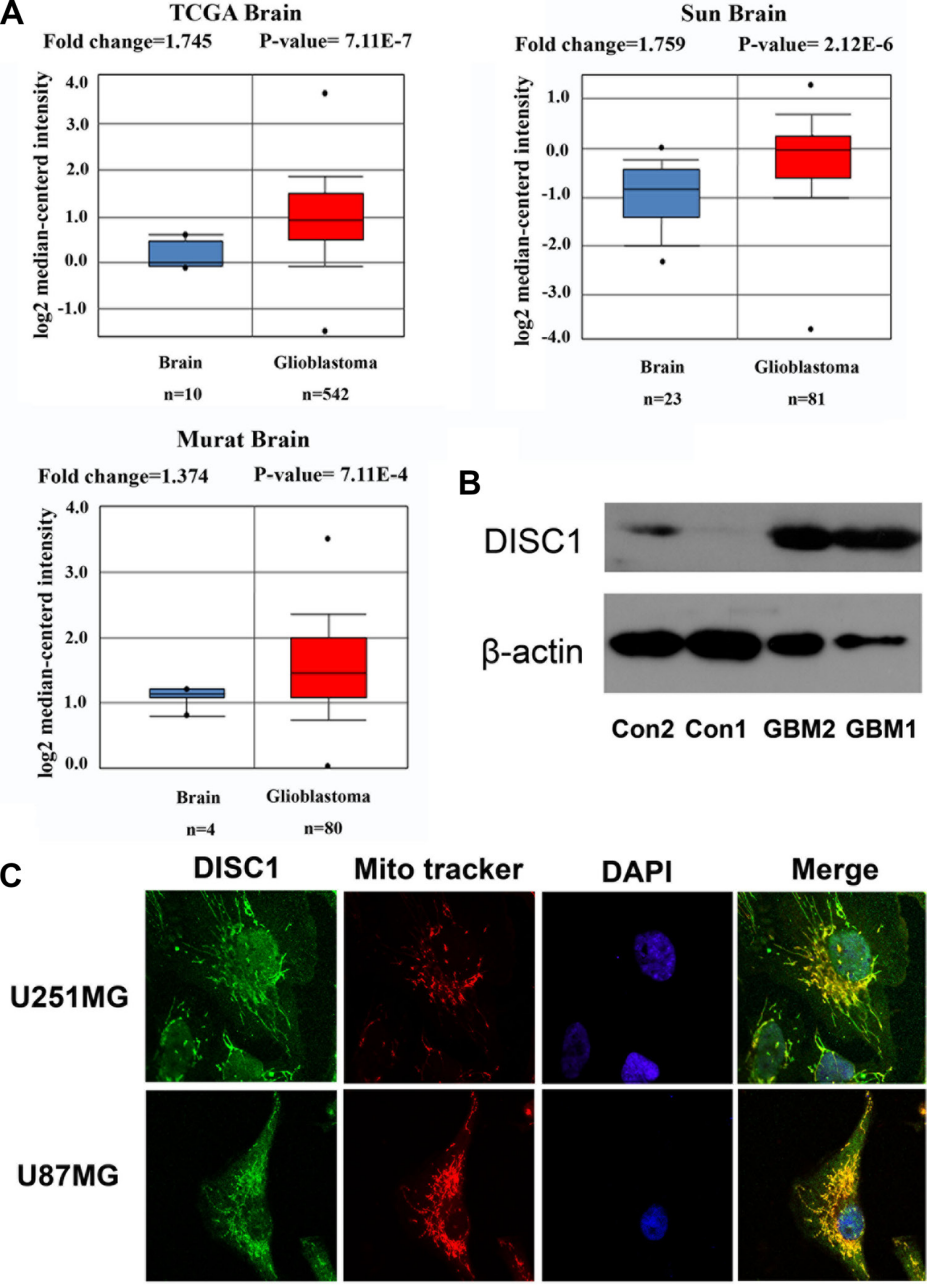
DISC1 expression in human glioblastoma (**A**) DISC1 expression in human glioblastoma in 3 independent studies culled from the Oncomine database (www.oncomine.org). Number of samples in each group and statistical significance of differences in expression levels are shown. (**B**) DISC1 expression in human glioblastoma and normal tissues. Con was adjacent normal tissue. (**C**) DISC1 localized in mitochondria and nuclear by immunofluorescence analysis.

In order to test whether DISC1 is crucial for human glioblastoma tumorigenesis, we examined the publicly available data at www.oncomine.org revealing 3 studies in which glioblastoma gene expression was examined. All studies showed statistically significant up-regulation of DISC1 expression in glioblastoma compared to the normal tissues (Figure 1A). Furthermore, the higher expression of DISC1 was detected in glioblastoma tissues than normal tissues (Figure 1B). It has been reported that endogenously expressed DISC1 localizes in several subcellular structures, such as mitochondria, nuclear and actin cytoskeleton [14]. Consistent with these observations, immunofluorescence analysis of U251MG and U87MG cells showed that DISC1 localized in mitochondria and nuclear (Figure 1C).

### DISC1 inhibition by shRNAs reduces glioblastoma cell proliferation *in vitro* and *in vivo*

**Figure 2:**
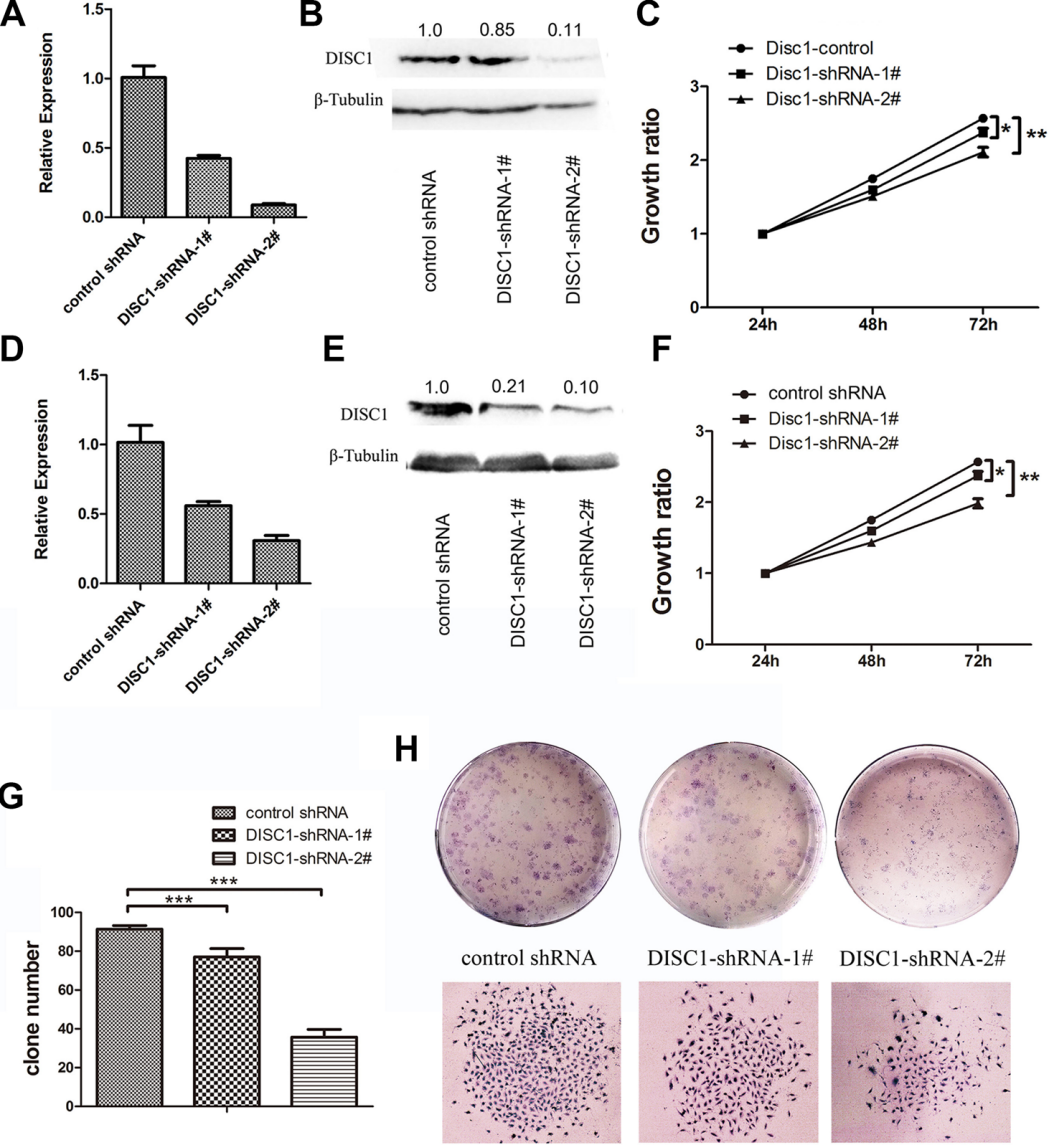
Reduced DISC1 inhibits glioblastoma cell proliferation *in vitro* and *in vivo* (**A**) qRT-PCR analysis detected the DISC1 expresssion in U87MG cells with endogenous DISC1 suppressed with shRNAs. (**B**) Western blotting analysis of DISC1 expression level in U87MG cells with endogenous DISC1 suppressed with shRNAs. The number showed the relative protein expression. (**C**) Effects of DISC1 knockdown with two independent lentiviral shRNA constructs on cell proliferation in U87MG. (**D**) qRT-PCR analysis detected the DISC1 expresssion in U251MG cells with endogenous DISC1 suppressed with shRNAs. (**E**) Western blotting analysis of DISC1 expression level in U251MG cells with endogenous DISC1 suppressed with shRNAs. (**F**) Effects of DISC1 knockdown with two independent lentiviral shRNA constructs on cell proliferation in U251MG. (**G**) Quantitation of colony number in each groups. (**H**) Representative images of colony formation for U251MG. Error bars represent SEM.*P < 0.05, **P < 0.01, ***P < 0.001.

To gain further insight into the function of DISC1 in tumor cells, DISC1 was knocked down in U87MG and U251MG cells. The knockdown effect of shRNA was tested by qRT-PCR and western blotting analysis in U87 MG cells (Figure 2A and 2B) and U251 MG cells (Figure 2D and 2E). The results showed that both two shRNAs could suppress the DISC1 expression, but the effect of DISC1-shRNA-2# was better than DISC1-shRNA-1#. WST-1 cell viability assays showed that down-regulation of DISC1 inhibited cell proliferation in both U87MG and U251MG cells (Figure 2C and 2F), and consistently, the inhibit effect of DISC1-shRNA-2# was better than DISC1-shRNA-1#. Furthermore, colony formation assay showed that the number of colonies was significantly decreased and the size of colony was smaller in U251MG cells depleted of DISC1 compared with the control (Figure 2G and 2H).

**Figure 3:**
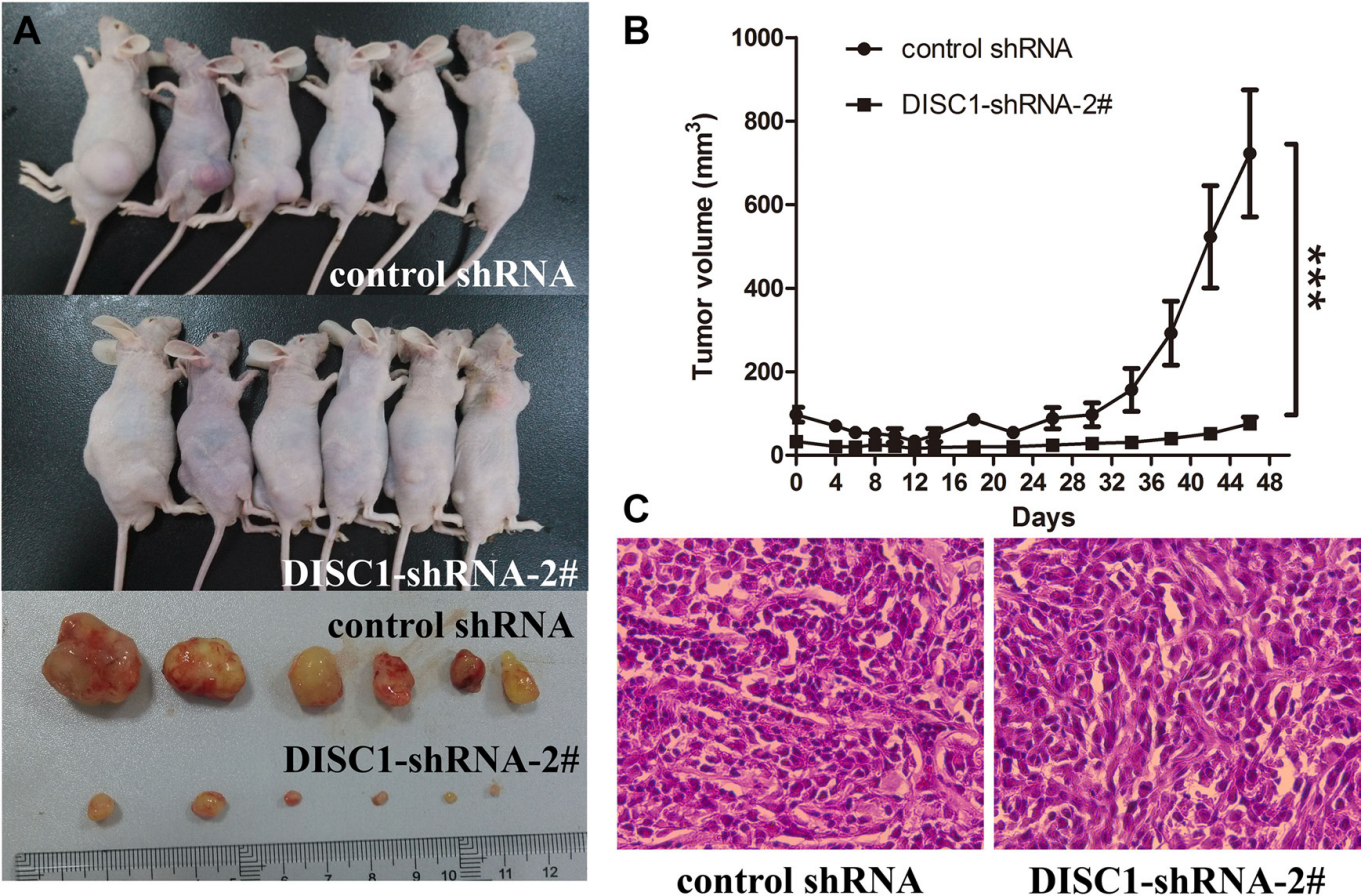
Significantly decreased tumor growth by DISC1 shRNAs in a xenograft model (**A**) Photographs of tumorigenesis in U87MG cells in xenograft mice and excised tumors. (**B**) Volume of xenograft tumors was measured at the indicated time points. (**C**) H&E staining of xenograft tumor tissues. Data are expressed as mean ± s.d. (*n* = 6). (C) H&E staining of xenograft tumor tissues. Magnification × 40.

To analyze the role of DISC1 in glioblastma carcinogenesis, we further assessed the effects of DISC1 on tumor growth *in vivo*. DISC1-shRNA-2#-U87MG cells and their respective control cells were implanted into the right and left flanks of nude mice by subcutaneous injection. At a postmortem examination conducted after 46 days, we found that tumors derived from DISC1-shRNA-2#-U87MG cells were significantly smaller than those originating from miR-control-U87MG cells (*n* = 6, animals per group) (Figure 3A and 3B). H&E staining showed decreased cell density in DISC1-shRNA-2#-U87MG xenografts (Figure 3C). Thus, knockdown of DISC1 significantly inhibited the proliferation of glioblastoma cells both *in vitro* and *in vivo*.

### shRNA-mediated knockdown of DISC1 inhibits U251MG cell migration and invasion

**Figure 4:**
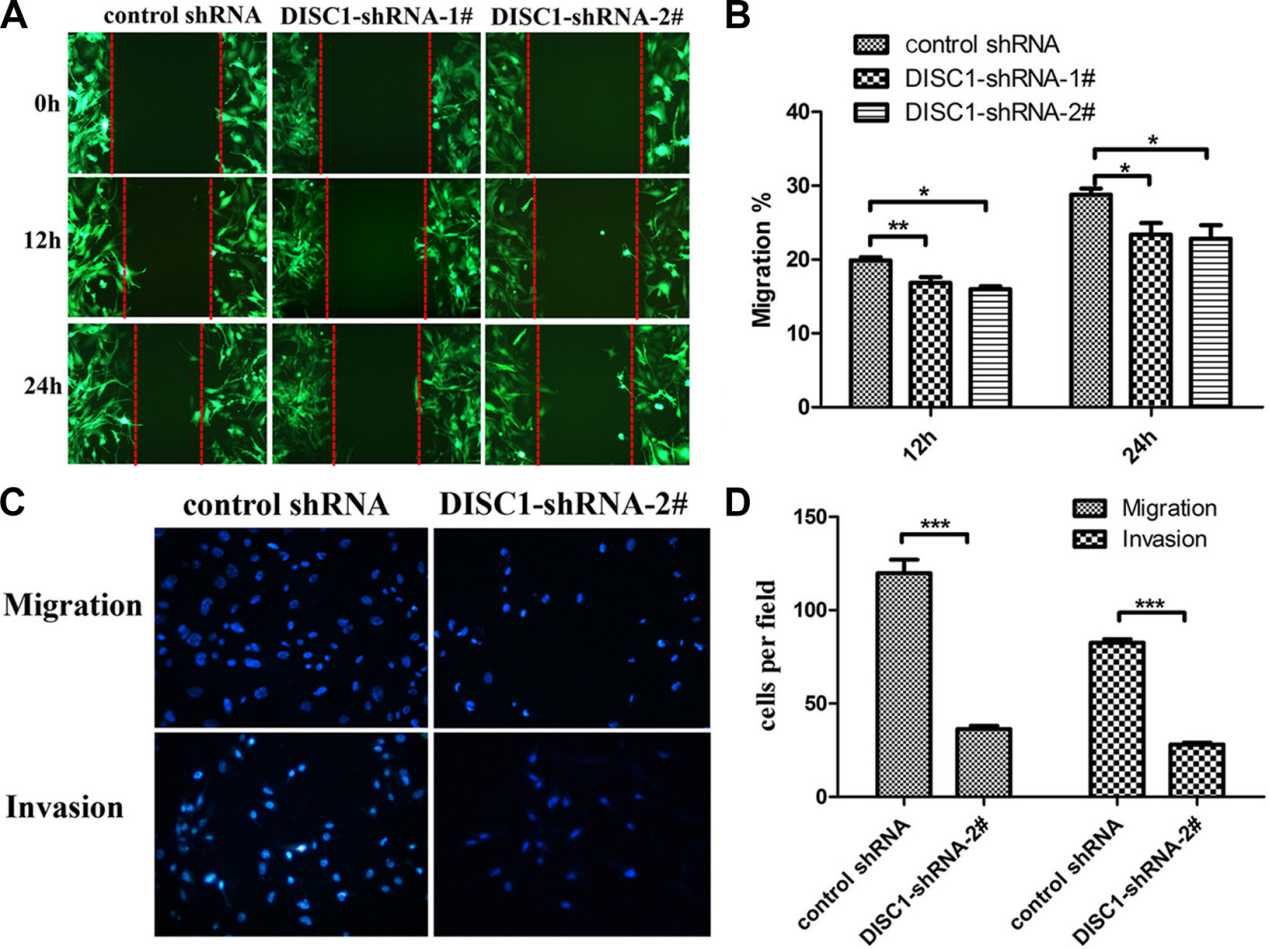
Knockdown of DISC1 inhibits the glioblastoma cell migration and invasion (**A**–**B**) The wound healing assay showed different cell migration in control-shRNA-U251MG, DISC1-shRNA-1#-U251MG and DISC1-shRNA-2#-U251MG. (A) Representative images were taken at different time points. (B) Quantification of cell motility by measuring the wound width. The amount of motility was expressed as a percent of migration at the zero time point. (**C** and **D**) Transwell assay of U251MG cells transfected with DISC1shRNA or negative control. (C) representative fields of invasive cells and migratory cells on the membrane. (D) quantitative analysis of the invasive and migratory cells from three independent experiments. Error bars represent SEM.*P < 0.05, **P < 0.01, ***P < 0.001.

To further investigate that whether DISC1 influences glioblastoma cell migration and invasion, wound-healing assay and transwell migration assay were performed. As shown in Figure 4A and 4B, compared with control cells, shRNA-U251MG cells, DISC1-shRNA-U251MG cells showed considerably slower migration. Furthermore, Transwell migration assays revealed that knockdown of DISC1 significantly restrained glioblastoma cell invasion (Figure 4C and 4D).

### shRNA-mediated knockdown of DISC1 inhibits the self-renewal capacity of glioblastoma stem-like cells

**Figure 5:**
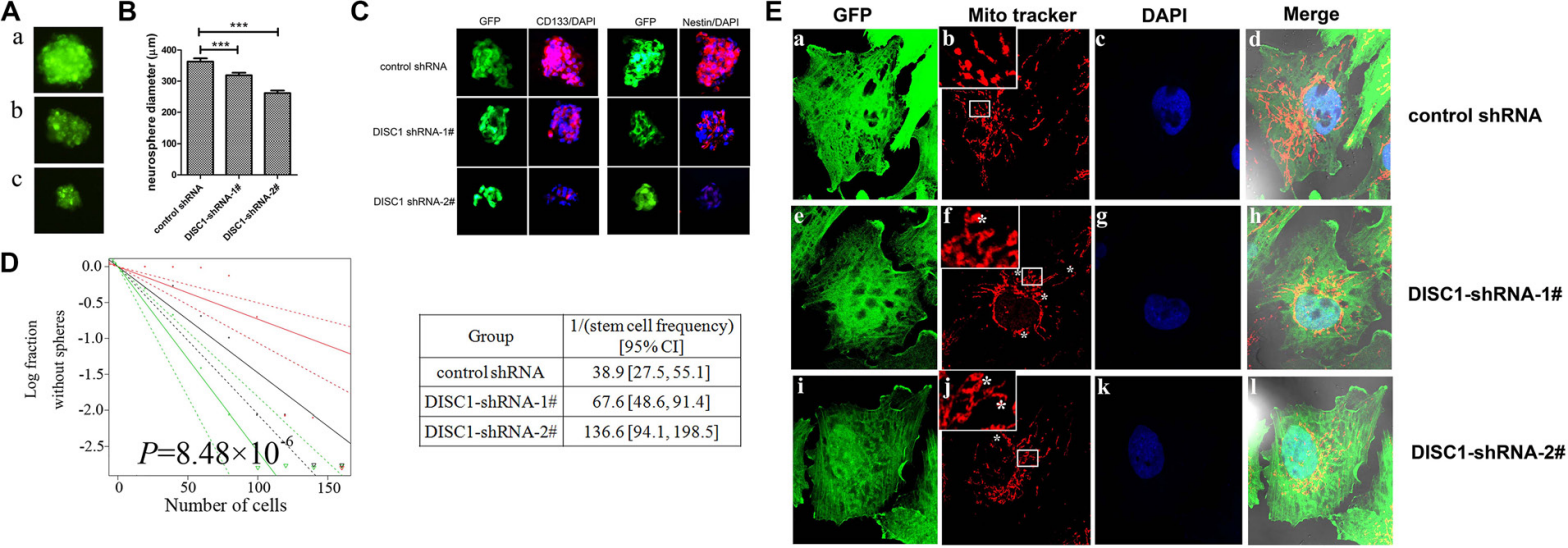
Targeting DISC1 by shRNA decreases glioblastoma stem-like cells self-renewal and alters the mitochondrial dynamic (**A**) The morphology of tumor spheres formed by the cancer stem cells from control-shRNA-U251MG (a) DISC1-shRNA-1#-U251MG(b) and DISC1-shRNA-2#-U251MG cells(c). (**B**) Glioblastoma neurosphere diameters decreased in DISC1-shRNAs U251MG cancer stem cells. (**C**) immunofluorescence images showing expression levels of nestin(red) and CD133(red) in neurospheres derived from control-shRNA-U251MG, DISC1-shRNA-1#-U251MG and DISC1 shRNA-2#-U251MG. cells. DAPI (blue) was used to stain nuclei. Scale bar, 200 μm. (**D**) *In vitro* extreme limiting dilution assays to single cells demonstrate that knockdown of DISC1 in U251MG cells decreases the frequency of tumorsphere formation (*P* = 8.48 × 10^−6^ by ANOVA). (**E**) Immunofluorescent imaging of DISC1 within U251MG cells. Inserts shows enlarged section for clarity. a lariat-like structure or ring mitochondria marked with stars (*).

The effect of DISC1 on glioblastoma cell self-renewal was further examined. U251MG cells were cultured in serum free medium (SFM) and tumor sphere formation was observed. As shown in Figure 5A and 5B, we found a significant decrease in the diameter of U251MG neurospheres in DISC1 knockdown groups (318.9 ± 53.5 μm of DISC1 shRNA-1#-U251MG spheres and 261.6 ± 52.8 μm of DISC1 shRNA-2#-U251MG spheres), compared to that of controls (362.9 ± 68.0 μm of control-shRNA-U251MG spheres). Next, We examined stem cell markers CD133 and nestin in these neurospheres by immunofluorescence staining, and found that DISC1 shRNA U251MG derived neurospheres showed decreased CD133 and nestin compared to control shRNA U251MG neurospheres (Figure 5C).

The self-renewal capacity of cancer stem-like cells in the U251MG cells was detected with a limiting dilution assay. The number of cells required to generate at least one tumor sphere/well was calculated as 67.6 in DISC1 shRNA-1#-U251MG cells, 136.6 in DISC1 shRNA-2#-U251MG and 38.9 in control-shRNA-U25MG cells (Figure 5D). All the data proposed the hypothesis that down-expression of DISC1 reduced the glioma stem-like cell stemness.

### Knockdown of DISC1 alters the mitochondrial dynamic by regulating Drp1

We further investigated whether the reduction of DISC1 would affect the mitochondrial morphology of glioblastoma cells. Mitochondria morphology in U251MG cells infected by lentivirus mediated DISC1 shRNA became abnormal compared to control shRNA (Figure 5E). In DISC1-shRNA U251MG cells, the mitochondria formed multiple independent rings or spheres in comparison to the normal filamentous morphology in control shRNA cells (Figure 5Ee, and 5Ej). In DISC1-shRNA-2#-U251MG cells, ring or lariat-like structures or mitochondria with a hole were also observed (Figure 5Ej).

**Figure 6:**
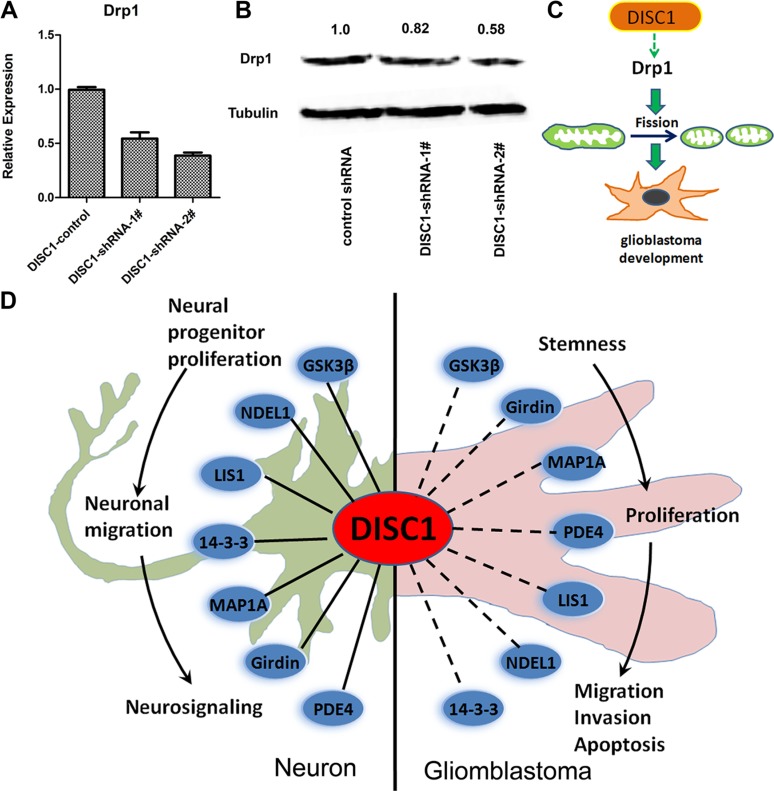
DISC1 down-regulation inhibits Drp1 expression and The ‘DISC1 interactome’ points towards the multiple functions of DISC1 during neuronal and glioblastoma development (**A**) qRT-PCR analysis detected the Drp1 expresssion in U251MG cells with endogenous DISC1 suppressed with shRNAs. (**B**) Western blotting analysis of Drp1 expression level in U251MG cells with endogenous DISC1 suppressed with shRNAs. The number showed the relative protein expression. (**C**) The illustration depicts the DISC1 inhibits glioblastoma development by regulating mitochondrial dynamics through promoting Drp1 expression. (**D**) The important DISC1 interactions both regulate neuronal and gliomblastoma development are highlighted in this figure. The solid line represents the interactions have been proved in neuron, the dotted line represents the interactions haven’t been proved in gliomblastoma, these were inferred from the neuron interactions. DISC1, Disrupted-in-schizophrenia-1; GSK3β, Glycogen synthase kinase 3β; NDEL1, Nucleardistribution protein nudE-like 1; LIS1, Lissencephaly protein 1; PDE4; Phosphodiesterase type 4; MAP1A, Microtubule-associated protein 1A.

Mitochondria are highly dynamic structure, undergoing constant fission and fusion which are essential for maintaining cell functions. Dynaminrelated protein 1 (Drp1) was reported to be important in the regulation of mitochondrial dynamics. We observed a significant down-regulation of Drp1 after DISC1 inhibition in U251 MG cells by both qt-PCR and western blotting (Figure 6A–6B).

## DISCUSSION

Glioblastoma is one of the most lethal brain cancers, and DISC1 is found to be up-regulated in glioblastoma through systematic bioinformatic analyses, suggesting that DISC1 may play a role in GBM tumorigenesis. Indeed, our results showed that reduced DISC1 by shRNA could suppress glioblastoma cell proliferation *in vitro* and *in vivo*, inhibit glioblastoma migration, invasion and glioblastoma stem-like cells self-renewal. Furthermore, we found that knockdown of DISC1 led to ring or lariat-like mitochondria and reduced the Drp1 expression, indicating a role of DISC1 in mitochondrial fusion and/or fission.

Mitochondria play important roles in central nervous system and cancer development. For a long time, defects in mitochondrial function have been suspected to contribute to the development and progression of cancer and detrimental consequences for brain development and function. It has been reported that DISC1 localizes in mitochondria and plays essential role for mitochondrial function in collaboration with Mitofilin [14, 15]. DISC1 also associates with Miro1 and TRAK1 to modulate anterograde axonal mitochondrial trafficking, since that DISC1 point mutations cause disrupted mitochondrial trafficking[16]. Recently, Norkett (2016) demonstrates that DISC1 couples to Miro1, Miro2, TRAK1 and TRAK2 to affect mitochondrial trafficking in axons and dendrites, and shows that DISC1 is a part of a native complex with Mitofusin1 and DISC1-Boymaw. Fusion protein decreases mitochondrial fusion, proving that DISC1 controls the morphogenesis of complex neuronal dendrites by regulating mitochondrial dynamics [17]. Our results showed DISC1 regulated the mitochondrial dynamics by regulating Drp1 expression. Thus, DISC1 is an important regulator of mitochondrial function in neuronal development. However, the mechanism of DISC1 regulating mitochondrial functions in tumor need to be clarified.

Mitochondrial dynamic is dependent on the balance of fission and fusion, aberrant mitochondrial dynamics are associated with major mental illness and tumorigenesis [18–28]. It has been reported that over expression of truncated DISC1 in the C-terminus caused formation of ring-like structures. We also proved this event in glioblastoma cells, that knockdown of DISC1 by shRNA produced ring or lariat-like mitochondria (Figure 5E), indicating that DISC1 participated in mitochondrial fusion and/or fission. Mitochondrial fusion is regulated by Mitofusion1/2 at the outer membrane of mitochondria, and OPA1 at the inner membrane [29]. Fission is coordinated by Drp1 and anchors such as Fis1 (mitochondrial fission protein 1), Mff (mitochondrial fission factor). It has been reported that Drp1 is upregulated in human invasive breast carcinoma and metastases to lymph nodes, and silencing Drp1 or overexpression of Mfn1 inhibit lamellipodia formation, a key step for cancer metastasis, suggesting that mitochondrial dynamics regulates migration and invasion of breast cancer cells [25]. In human glioblastoma U251 cells, Drp1 has been reported to be involved in hypoxia-induced migration [30]. We also reported here that shRNA-mediated knockdown of DISC1 inhibited glioblastoma cell migration and invasion (Figure 4), indicating that mitochondrial dynamics had an important role in gliomblastoma migration and invasion, and DISC1 regulated the mitochondria dynamics in part by blocking Drp1 (Figure 6C). Recently, yin *et al*. reports that Drp1 is highly expressed in glioma tissues, and silencing Drp1 inhibits glioma cells proliferation and invasion by RHOA/ROCK1 pathway. Thus it confirms that DISC1 -facilitates glioma developement by up-regulation of Drp1.

As known, DISC1 regulates multiple critical pathways in the developing and adult brain, and we proved that DISC1 could regulate gliomblastoma, which suggesting that DISC1 had dual effect of regulating neurodevelopment and gliomblastoma tumorigenesis. In 2007, Camargo and colleagues disclose of the ‘DISC1 interactome’, a complex protein-protein interaction network based off yeast-two hybrid screens using DISC1 and a set of DISC1 interactors as baits [31]. Understanding the biology of DISC1-interacting proteins and the functions of these protein complexes will help us understanding DISC1’s biological function; we summarized the interacting proteins which played a role in neuronal development and tumor glioma development in Figure 6D. It has been reported that GSK3β, NDEL1, LIS1, 14-3-3, MAP1A, Girdin and PDE4 interact with DISC1 to regulate the neural progenitor proliferation [32], neuronal migration [33, 34] and neuronal signaling [35, 36]. All these proteins have been reported to play an important role in glioma development. GSK3β regulates glioblastoma cell invasion, apoptosis and stem cell stemness [37, 38]; LIS1 and NDEL1 play a role in glioma migration and proliferation analogous to their role during brain development [39];14-3-3 regulates glioma cell proliferation and apoptosis [40, 41]; MAP1A is strongly expressed in high grade glioma and may play an important role in cell proliferation [42]; Girdin is reported to be involved in glioblastoma cell migration, adhesion, invasion and stem cell stemness [43–45]; PDE4 inhibitors have been reported to suppress glioblastoma growth *in vitro* and *in vivo* [46]. Until now, there is no research on DISC1 and gliomblastoma, since the DISC1-interacting proteins play important roles in gliomblastoma, and our results prove that DISC1 regulates gliomblastoma development, so we propose that DISC1 may be as an interactome in regulating gliomblastoma tumorigenesis.

Finally, we have reported evidence of interplay between DISC1 and tumorigenesis. For the first time, our data show that DISC1 have an important role in glioblastoma cell proliferation, migration, invasion and cancer stem-like cell self-renewal by regulating mitochondrial dynamics via Drp1. DISC1 has been proved to be a key regulatory molecule in diverse processes of neurodevelopment, and pediatric gliomas as reported as a neurodevelopmental disorders [47] so we think that DISC1, a gene which has already proved to be involved in regulating neurodevelopment, but also might be involved in regulating GBM tumorigenesis.

## MATERIALS AND METHODS

### Cell lines and cancer stem-like cell culture

Human U87MG and U251MG glioblastoma cell lines were purchased from the Chinese Academy of Sciences Cell Bank in 2015. The authenticity of cancer cell lines was tested by short tandem repeat profiling (STR). All cell lines were grown in DMEM medium supplemented with 10% FBS (GIBCO) and 1% NEAA (GIBCO). The serum free medium (SFM) was composed of DMEM/F12, 20 ng/mL basic fibroblast growth factor (bFGF; peprotech), 20 μL/mL B27 supplement (Life Technologies), and 20 ng/mL EGF (peprotech). Glioma stem-like cells (GSC) were isolated from U251 MG glioblastoma cell lines by using SFM. These cells can form neurosphere-like cell aggregates in less than 7 days (17).

### Oncomine analysis and tumor specimens

Oncomine (Compendia Bioscience, Ann Arbor, MI) was used for analysis and visualization of the glioblastoma (http://www.oncomine.org). DISC1 RNA expression levels were displayed using log2 median centered ratio boxplots for GBM vs brain. 2 samples of glioblastoma (grade IV) and 2 adjacent normal tissues were collceted from affiliated hospital of Xi’an Medical University, the local Ethical Committee approved our study, and all patients provided informed consent.

### Proliferation and colony formation assay

Cells were seeded at a density of 5000 cells per well in 96-well plates and and incubated for 24 h, 48 h, 72 h, respectively. An aliquot of 10 μL of CCK-8 was added to the wells and incubated for 1 h (Beyotime, Shanghai, China). The absorbance was measured at 450 nm to calculate the numbers of viable cells in each well. Each measurement was performed in triplicate and the experiments were repeated twice.

For colony formation assays, cells were seeded in six-well plates at a density of 200 cells per well and cultured at 37°C for two weeks. At the end of the incubation, the cells were fixed with 100% methanol and stained with 0.1% (w/v) Crystal Violet. Megascopic cell colonies were counted using Image-Pro Plus 5.0 software (Media Cybernetics, Bethesda, MD, USA). Each measurement was performed in triplicate and the experiments were each conducted at least three times.

### Animal studies

U87MG cells stably expressing DISC1-shRNA-2# or empty vector controls were implanted in the flanks of athymic mice (3.0×10^6^/200 μl per mice, total 6 mice). Tumor volumes were determined by measuring the length (a) and the width(b). The tumor volume (V) was calculated according to the formula V = (ab)^2^/2. All mouse experiments were performed in accordance with institutional guidelines and regulations of the government.

### Wound healing assays

U251MG cells were seeded in 6-well plates and cultured until confluence. A wound was then created by manually scraping the cell monolayer with a 200 mL pipette tip. The floating cells were removed by washing twice by PBS. Then cells were incubated in DMEM supplemented without FBS. Cell migration into the wound was observed at three preselected time points (0, 12, and 24 hours) in six randomly selected microscopic fields for each condition and time point. Images were acquired with a Nikon DS-5M Camera System mounted on a phase-contrast Leitz microscope and were processed using Adobe Photoshop 7.0. The distance traveled by the cells was determined by measuring the wound width at different time points and then subtracting it from the wound width at time 0. The values obtained were expressed as a migration percentage, setting the gap width at 0 hour as 0% [48, 49].

### *In vitro* migration and invasion assays

Cells (5×10^5^) were planted on the top side of polycarbonate Transwell filters (without Matrigel for Transwell assay) or plated on the top side of polycarbonate Transwell filter coated with Matrigel(for Transwell matrix penetration assay) in the upper chamber of the QCM™ 24-Well Cell Invasion Assay (Cell Biolabs, INC, USA & Canada). For transwell migration assays, cells were suspended in medium without serum, and medium without serum was used in the lower chamber. For the invasion assay, cells were suspended in medium without serum, and medium supplemented with serum was used as a chemoattractant in the lower chamber. The cells were incubated at 37°C for 8 hours (transwell assay) or 48 hours (invasion assay). The non-migratory or non-invasive cells in the top chambers were removed with cotton swabs. The migrated and invaded cells on the lower membrane surface were fixed in 100% methanol for 10 min, air-dried, then stained with DAPI and counted under a microscope. Three independent experiments were performed and the data were presented as the means±standard error of mean (SEM).

### Quantitative RT-PCR

Total RNA was isolated using Trizol reagent (Invitrogen) and reverse-transcribed into cDNA using BcaBest RNA PCR kit from TaKaRa according to the manufacturer’s instructions. Quantitative real-time PCR was performed using a Peltier Thermal Cycler (BioRad) plus Realtime PCR Master Mix (SYBR Green, Toyobo, Osaka, Japan). The specific primers used for PCR are listed in Supplementary Table S1. GAPDH was chosen as the endogenous control in the assay.

### Western blotting

Cells were collected and lysed in high KCl lysis buffer with complete protease inhibitor cocktail (Roche)[48]. The protein concentration was determined using a BCA protein assay kit (Pierce). The samples were separated by SDS-PAGE and transferred to polyvinylidene fluoride membranes (Roche). The membranes were treated with 5% nonfat dry milk in TBS, followed by incubation with primary antibodies and then POD-labeled secondary antibodies (Roche). The immunolabeled proteins were detected using ECL detection system (Boster, Wuhan, China). Densitometry quantification was acquired with Gel Doc 1000 system and was analyzed using the Quantity One software. The following primary antibodies were used: DISC1 (abcam; 1:5000), β-tubulin (Santa Cruz; 1:2000), Drp1 (abcam, 1:2000).

### Immunofluorescence and hematoxylin and eosin staining

Cells on poly-L-lysine-coated glass cover slips were fixed with 4% paraformaldehyde for 15 min at room temperature and then permeabilized by treatment with ice-cold methanol for 10 min. Cancer spheres were fixed in 4% paraformaldehyde, embedded in optimal cutting temperature (OCT) compound for freezing, and then cryosectioned (15 μm sections). After being blocked by 15% normal donkey serum for 30 min, the cells were incubated at room temperature for 1 h with primary antibody diluted in antibody buffer (50 mM Tris-HCl, pH 7.4, 150 mM NaCl, 100 mM L-Lysine, 1% BSA and 0.04% azide). The following antibodies were used: CD133 (Elabscience, 1:100) and nestin (abcam, 1:100). After incubation with the primary antibodies, the cells were rinsed and incubated for 1 h at room temperature with Alexa Fluor-labeled secondary antibodies (Molecular Probes, Leiden, The Netherlands; 1:800). The cells were washed with PBS and the cover slips were mounted with glycerine/PBS containing 5 mg/ml DAPI for nuclei staining.

To determine subcellular distribution of mitochondria, cells were loaded with 50nm MitoTracker Red (Life Technologies Corporation, Grand Island, NY, USA) for 30 min to stain mitochondria. Images were taken using a Leica confocal.

For H&E staining, we embedded the fixed tumor in paraffin, cut them into 6-μm sections and stained with H&E. Slides were photographed using an optical or confocal microscope (OLYMPUS).

### Limiting dilution assay

For limiting dilution assays, cells after puromycin selection were counted and with decreasing numbers of cells per well (160, 140, 120, 100, 80, 60, 40, 20) plated in 96-well plates. Fourteen days after plating, the presence and number of tumorspheres in each well was quantified. Extreme limiting dilution analysis was performed using software available at http://bioinf.wehi.edu.au/software/elda/, as previously described [50].

### Statistics

Data were analyzed using the two-tailed Student’s *t*-test. *P* < 0.05 was considered statistically significant.

## SUPPLEMENTARY TABLE


