# Supplementary material for: Disrupted in schizophrenia 1 (DISC1) inhibits glioblastoma development by regulating mitochondria dynamics
Source: Oncotarget. 2016 Nov 11;7(52):85963–74. doi: 10.18632/oncotarget.13290 (PMC5349889; doi:10.18632/oncotarget.13290)
Supplement: Supplementary file 1 [file oncotarget-07-85963-s001.pdf]

Disrupted in schizophrenia 1 (DISC1) inhibits glioblastoma development by regulating mitochondria dynamics

Supplementary Materials

Supplementary Table S1: Primer and shRNA sequences used in the study

primer name	sequence
GAPDH-F	GAAGGTGAAGGTCGGAGTC
GAPDH-R	GAAGATGGTGATGGGATTTC
DISC1-DR	AACCCTACCTACTTCCGGCT
DISC1-WR	GCTATTGCTGCCCGGGGTAC
hDRP1F	GATGCCATAGTTGAAGTGGTGAC
hDRP1R	CCACAAGCATCAGCAAAGTCTGG
shRNA name	sequence
control shRNA	TTCTCCGAACGTGTCACGT
DISC1 ShRNA-1#	GCAGTTGAGAATGATGATTAT
DISC1 ShRNA-2#	GCAGGAGGTCAGCAAGGCCTTG
